# Recalibration and external validation of vascular risk calculators for multiple sclerosis: A population-based study using CPRD data in England, 1987–2023

**DOI:** 10.1177/13524585261444243

**Published:** 2026-05-12

**Authors:** Raffaele Palladino, Ruth Ann Marrie, Giuseppina Affinito, Azeem Majeed, Stephen Allan Schaffer, Jeremy Chataway

**Affiliations:** Department of Primary Care and Public Health, School of Public Health, Imperial College of London, London, UK; Department of Public Health, Federico II University, Naples, Italy; Departments of Medicine and Community Health and Epidemiology, Dalhousie University, Nova Scotia Health, Halifax, NS, Canada; Nova Scotia Health Authority, Halifax, NS, Canada; Department of Public Health, Federico II University, Naples, Italy; Department of Primary Care and Public Health, School of Public Health, Imperial College of London, London, UK; Department of Internal Medicine, Faculty of Health Sciences, University of Manitoba, St Boniface General Hospital, Winnipeg, MB, Canada; Queen Square Multiple Sclerosis Centre, Department of Neuroinflammation, UCL Queen Square Institute of Neurology, Faculty of Brain Sciences, University College London, London, UK; National Institute for Health and Care Research, University College London Hospitals Biomedical Research Centre, London, UK

**Keywords:** Multiple sclerosis, vascular, cardiovascular, recalibration, risk, population-based study

## Abstract

**Background:**

People with multiple sclerosis (PwMS) have an elevated risk of macrovascular disease that may be underestimated by vascular risk calculators (VRCs) validated in the general population.

**Objectives:**

This study evaluated, recalibrated and externally validated five commonly used VRCs for PwMS using population-based data from England, 1987–2023.

**Methods:**

PwMS and matched controls were identified from Clinical Practice and Research Datalink (CPRD) GOLD (calibration) and CPRD Aurum (validation). Exposure variables included multiple sclerosis (MS) status and risk factors as defined in Atherosclerotic Cardiovascular Disease, Framingham Risk Score (FRS), FRS-BMI (body mass index), QRESEARCH risk estimator version 3 score and Systematic Coronary Risk Evaluation version 2 score (SCORE2). Model performance was assessed using Somers’ D statistic, area under the receiver operating characteristic (ROC) curve and Hosmer–Lemeshow chi-square test. VRCs with ROC < 0.70 in PwMS were recalibrated using Cox regression, incorporating MS status. Ten-fold cross-validation was used to estimate Somers’ D.

**Results:**

Calibration: 9411 PwMS and 57,805 controls; validation: 45,934 PwMS and 278,452 controls. Discrimination declined with standard thresholds (e.g. SCORE2 sensitivity in PwMS, 30.0%). Only FRS-BMI retained all significant predictors and was successfully recalibrated, improving discrimination (Somers’ D = 0.815 vs. 0.792; Δ = 0.023) and showing good calibration. External validation showed modest gain (Somers’ D = 0.716; Δ = 0.003).

**Conclusion:**

These findings underscore the limitations of general-population VRCs in PwMS and support the development of MS-specific vascular risk models.

## Introduction

People with multiple sclerosis (PwMS) experience a heightened risk of cardiovascular and cerebrovascular disease,^1^ which is not entirely accounted for by traditional vascular risk factors such as hypertension, hyperlipidaemia, diabetes and smoking.^1,2^ Other possible contributing factors may include accelerated biological ageing, systemic inflammation, endothelial dysfunction, prothrombotic and autonomic mechanisms, cerebral hypoperfusion, genetic liability, treatment exposure and comorbidity.^3–5^ These risk factors contribute to premature mortality or accelerated disability progression.^4^

However, despite their importance, vascular comorbidities are underdiagnosed and undertreated in PwMS.^6^

Clinical guidelines recommend the use of vascular risk calculators (VRCs) to stratify individuals based on their 10-year risk of vascular events, enabling targeted preventive interventions. Commonly used tools, including the Framingham Risk Score (FRS), BMI-based Framingham Risk Score (FRS-BMI),^7,8^ QRESEARCH risk estimator version 3 score (QRISK3),^9^ Systematic Coronary Risk Evaluation version 2 score (SCORE2)^10^ and the Atherosclerotic Cardiovascular Disease (ASCVD) score,^11^ were validated in general populations and may not accurately estimate vascular risk in PwMS. In rheumatoid arthritis, another chronic inflammatory disease, VRCs underestimate cardiovascular risk in low- and medium-risk categories, while overestimating it in high-risk groups.^12^ These findings suggest that systemic inflammation and disease-specific characteristics influence vascular risk in ways that are not accounted for by standard VRCs.

Furthermore, age is a key determinant of vascular risk scores.^7–11^ Multiple sclerosis (MS) is frequently diagnosed at a mean age of 30 years globally;^13^ so PwMS are systematically attributed low absolute risk, even when other clinical risk factors are present. This structural bias means that younger PwMS with subclinical or emerging vascular comorbidities will be excluded from early interventions. Therefore, recalibration of existing VRCs is needed for PwMS, as was also demonstrated with the WHO’s Fracture Risk Assessment Tool (FRAX®).^14^

We aimed to (1) evaluate the performance of five commonly used VRCs: FRS, FRS-BMI, QRISK3, SCORE2 and ASCVD in PwMS, compared to people without MS; (2) recalibrate each VRC using population-level data and (3) externally validate those recalibrated VRCs that demonstrated improved/good performance using population-level data from an external data set of PwMS.

## Methods

We conducted a population-based study of PwMS and matched controls registered with general practices in England, from 1987 to 2023. The study used data from routinely collected electronic health records from two Clinical Practice and Research Datalink (CPRD) databases. Ethics approval was granted by the Independent Scientific Advisory Committee of the CPRD (protocol numbers: 22_002520 and 18_279R).

### Data source

We used two of the largest UK electronic health record databases: CPRD GOLD (1986–2018) and CPRD Aurum (1990–2023), which hold anonymized, routinely collected longitudinal primary care records.^15,16^ They are representative in terms of socio-demographic characteristics.^15–18^ The *calibration* cohort was drawn from CPRD GOLD, which includes data from general practices using the Vision® software system. The *validation* cohort was drawn from CPRD Aurum. For both data sources, we limited the analysis to English practices^15^ as primary care data are linked to multiple sources, including Hospital Episode Statistics (HES), Office for National Statistics (ONS) mortality and deprivation data, using National Health Service numbers.

### Study population

MS cases were identified using a previously described algorithm (supplemental methods).^1^ The index date was considered the date of the first MS diagnosis.^1^ Inclusion criteria were the same as for prior work (see supplemental methods).^1^ To enhance sample size, we included up to two time points per participant: data from the index year and, when available, data collected at age 50 (±5 years). This age range was selected given that vascular risk assessments are commonly performed around age 50 years in England.^19^

To assess the discriminative performance of the VRCs, PwMS were randomly matched to as many as six individuals without MS based on general practice, sex and age. Controls were required to have up-to-standard clinical data available during the study period (CPRD Gold only) and to have no recorded diagnosis of MS or any other demyelinating condition (e.g. optic neuritis, transverse myelitis, acute disseminated encephalomyelitis or central nervous system demyelination not otherwise classified); this reduced the likelihood of including individuals who might later develop MS. Multiple controls were matched to each PwMS to decrease variance. We assigned the controls the index date of their matched MS case.

As VRCs estimate the 10-year risk of cardiovascular disease (CVD) or CVD-related mortality, we excluded individuals with a history of these outcomes before the index date, tailoring the inclusion criteria to match the specific definitions of CVD and applicable age ranges used by each calculator (see Supplemental Table 1).

### Study variables

All variables considered for this study included age; sex; ethnicity; index of multiple deprivation;^20^ BMI; smoking status; systolic blood pressure (SBP) and a measure of SBP variability; total cholesterol and total cholesterol/high-density lipoprotein (HDL) ratio; diagnosis of the following chronic diseases: diabetes, chronic renal disease, migraine, lupus and severe mental illness; treatment with antihypertensive medication, corticosteroid and atypical antipsychotics; and family history of macrovascular disease. Outcomes included the 10-year risk of CVD and CVD-mortality. As part of the CVD definition (see Supplemental Table 1 for details), cerebrovascular disease was identified from CPRD, HES and ONS records using validated, primary care records, stroke-specific ICD-10 codes in hospital data and cause-of-death codes where applicable, an approach that improves completeness and accuracy of stroke capture in English routine data.^21–23^ Supplemental Table 1 summarizes the variables included in each VRC.

### Statistical analysis

PwMS with missing data on smoking at index were classified as non-smokers if there was no prior indication of smoking history.^24^ Those missing data on ethnicity were classified as White.^1^ For BMI, SBP, total cholesterol and HDL, if data were missing at the index year, we considered records in the following and previous 5 years.^1^ Consistent with previous studies using CPRD data in MS research,^1^ we employed the multiple imputation method to handle missing data for time-varying variables, including BMI, SBP, total cholesterol and HDL. After checking the missing data percentage for these variables and multiple imputation assumptions, we employed multiple imputation by chained equation (10 copies) and combined results using Rubin’s rules for BMI and SBP (calibration cohort: 49.9% for blood pressure and 50% for BMI; validation cohort: 24.9% for blood pressure and 25.7% for BMI).^25^ Given the high percentage of missing data, multiple imputation was not possible for cholesterol data. The regression model for the multiple imputation included these independent variables: sex, age, ethnicity, smoking status in the index year, number of comorbidities (considering those included in FRS, FRS-BMI, SCORE2, ASCVD and QRISK3)^8,26,27^ in the index year, treatment with lipid-lowering, oral anti-diabetic, anti-platelet, anti-coagulant and antihypertensive therapies in the index year, number of visits in the previous year, region and index of multiple deprivation (quintiles).^20^

The recalibration and validation process comprised several steps. First, we assessed the performance of the original risk equation employed for the VRCs in the calibration cohort by calculating the receiver operating characteristic (ROC) curve area using the Youden index approximation, considering the continuous score and the binary variable defined by the published cut-point for intervention (binary classifier) for each VRC. Second, for ROC < 0.70 for PwMS, we recalibrated each VRC, which involves adjusting baseline hazard functions and population-specific means to better reflect the target group, an approach applied elsewhere.^14^ Specifically, we employed Cox regression models including the terms of the original equations as independent variables and added MS status (binary variable) as an additional term.^28^ We limited model extension to the inclusion of an MS indicator to preserve real-world applicability since our recalibration was designed for implementation using routinely collected data, also in primary care settings where MS-specific measures such as phenotype, disability status, inflammatory activity and detailed disease-modifying therapy (DMT) exposures are not consistently available. Transformations applied to continuous variables in the original VRCs were preserved during the recalibration and incorporated into the Cox model accordingly. Although we refer to this process as ‘recalibration’, it more accurately reflects a model revision approach. Specifically, we extended the original VRC by including MS status as an additional predictor; this constitutes a hybrid between recalibration and derivation.^29^ Third, the recalibration process was conducted only for risk equations in which all terms remained statistically significant (*p* < 0.05) in the Cox model to ensure the reliability and interpretability of the recalibrated coefficients. Including non-significant predictors would have resulted in unstable or non-reproducible point estimates, potentially introducing bias or reducing generalizability.^29,30^ As previously suggested, we evaluated the calibration of the derived risk algorithm in our internal data set by stratifying the cohort by deciles of risk score and employing a Hosmer–Lemeshow test.^8^ Finally, k-fold (10-fold) cross-validation was employed to calculate the Somers’ D statistics.^31^ For VRCs that were successfully recalibrated, an interaction term between MS and other terms was also tested. The recalibrated VRCs were tested externally using the validation and calculating the Somers’ D statistics;^31^ for completeness, the ROC curve for the externally validated score was additionally reported in the Supplemental Material.

## Results

### Baseline characteristics of the calibration cohort

Sample size varied across sub-cohorts. The FRS-BMI sub-cohort included nearly the full calibration sample (9411 PwMS and 57,805 controls), reflecting minimal exclusions beyond those with prior macrovascular disease or age outliers. The SCORE2-based sub-cohort was the smallest cohort (761 PwMS and 5252 controls) (see Supplemental Figure 1). At index date, among PwMS, the mean age ranged from 46.0 to 52.7 years depending on the VRC sub-cohort. Smoking prevalence was higher among PwMS compared with controls across all subgroups. Mean BMI, SBP and lipid profiles were broadly comparable between groups, although PwMS showed marginally lower SBP (Table 1).

**Table 1.** Descriptive statistics of the calibration cohorts.

ASCVD						
	Both sexes		Males		Females	
	Matched controls	PwMS	Matched controls	PwMS	Matched controls	PwMS
*N*	5808	795	1877	254	3931	541
Score	8.5 (9.5)	6.4 (7.6)	12.3 (10.1)	9.3 (8.4)	6.7 (8.6)	5.1 (6.7)
Age	55.9 (9.5)	52.7 (8.8)	55.8 (9.4)	52.2 (8.7)	56.0 (9.5)	52.9 (8.8)
Non-White	8.9%	5.5%	7.7%	6.3%	9.5%	5.2%
Smoker	22.4%	29.2%	25.7%	36.2%	20.8%	25.9%
BMI	28.2 (5.6)	27.9 (5.9)	28.1 (4.6)	27.3 (4.9)	28.2 (6.0)	28.2 (6.3)
SBP	133.4 (16.4)	130.6 (16.2)	135.9 (16.1)	130.5 (14.6)	132.2 (16.4)	130.6 (17.0)
Total cholesterol	5.4 (1.1)	5.5 (1.1)	5.3 (1.1)	5.3 (1.2)	5.5 (1.1)	5.5 (1.1)
HDL	1.6 (2.6)	1.5 (0.5)	1.3 (0.9)	1.4 (0.5)	1.6 (3.1)	1.5 (0.4)
Diabetes	12.9%	13.6%	13.7%	15.0%	12.6%	12.9%
Framingham						
	Both sexes		Males		Females	
	Matched controls	PwMS	Matched controls	PwMS	Matched controls	PwMS
*N*	6256	974	2016	306	4240	668
Score	12.0 (11.1)	9.7 (9.5)	18.4 (13.8)	14.5 (11.9)	9.0 (8.0)	7.5 (7.3)
Age	53.2 (10.4)	49.0 (10.3)	53.5 (10.2)	49.2 (10.3)	53.0 (10.4)	48.8 (10.3)
Non-White	9.4%	6.5%	8.3%	7.5%	9.9%	6.0%
Smoker	23.3%	30.8%	26.6%	36.6%	21.8%	28.1%
BMI	28.2 (5.7)	28.1 (6.2)	28.2 (4.7)	27.4 (4.9)	28.2 (6.2)	28.5 (6.7)
SBP	132.2 (16.4)	128.9 (16.0)	135.2 (16.2)	130.4 (14.3)	130.7 (16.4)	128.2 (16.7)
Total cholesterol	5.4 (1.1)	5.4 (1.1)	5.3 (1.1)	5.3 (1.1)	5.4 (1.1)	5.4 (1.1)
HDL	1.5 (2.5)	1.5 (0.5)	1.3 (0.9)	1.3 (0.5)	1.6 (3.0)	1.5 (0.4)
Diabetes	12.5%	13.1%	13.1%	15.0%	12.1%	12.3%
Framingham BMI						
	Both sexes		Males		Females	
	Matched controls	PwMS	Matched controls	PwMS	Matched controls	PwMS
*N*	57,805	9411	17,280	2800	40,525	6611
Score	8.4 (9.5)	8.7 (9.7)	0.1 (0.1)	0.1 (0.1)	6.1 (6.8)	6.4 (6.9)
Age	46.1 (10.6)	46.0 (10.6)	46.9 (10.5)	46.8 (10.5)	45.8 (10.6)	45.7 (10.5)
Non-White	7.8%	5.6%	7.2%	6.4%	8.1%	5.3%
Smoker	29.4%	38.0%	31.3%	41.0%	28.6%	36.7%
BMI	26.3 (4.2)	25.9 (4.7)	26.6 (3.5)	25.8 (3.8)	26.2 (4.4)	25.9 (5.1)
SBP	125.8 (13.6)	125.4 (14.0)	129.6 (12.6)	128.3 (13.0)	124.3 (13.6)	124.2 (14.2)
Diabetes	4.8%	6.8%	5.9%	8.5%	4.3%	6.0%
QRISK3						
	Both sexes		Males		Females	
	Matched controls	PwMS	Matched controls	PwMS	Matched controls	PwMS
*N*	6638	1035	2111	319	4527	716
Score	1062.5 (873.8)	872.5 (789.9)	857.5 (661.7)	645.8 (527.8)	1157.0 (940.9)	972.7 (862.8)
Age	53.6 (11.7)	48.2 (11.4)	53.7 (11.4)	48.3 (11.0)	53.5 (11.9)	48.2 (11.6)
Non-White	9.4%	7.1%	8.5%	7.8%	9.8%	6.7%
Smoker	23.0%	30.8%	26.4%	37.0%	21.4%	28.1%
BMI	28.1 (5.8)	28.1 (6.2)	28.1 (4.8)	27.3 (4.8)	28.1 (6.2)	28.5 (6.7)
SBP	132.3 (16.5)	128.7 (16.1)	135.2 (16.2)	130.4 (14.3)	130.9 (16.5)	127.9 (16.8)
Total cholesterol	5.3 (1.1)	5.3 (1.1)	5.3 (1.1)	5.3 (1.1)	5.4 (1.1)	5.4 (1.1)
HDL	1.5 (2.5)	1.5 (0.5)	1.3 (0.9)	1.3 (0.5)	1.6 (2.9)	1.5 (0.4)
Diabetes	12.6%	12.9%	13.4%	15.0%	12.3%	12.0%
SCORE2						
	Both sexes		Males		Females	
	Matched controls	PwMS	Matched controls	PwMS	Matched controls	PwMS
*N*	5252	761	1722	246	3530	515
Score	4.1 (3.0)	3.7 (2.9)	5.7 (3.3)	5.0 (3.2)	3.3 (2.4)	3.1 (2.5)
Age	54.1 (7.9)	51.8 (7.8)	54.2 (8.1)	51.6 (8.1)	54.0 (7.8)	51.8 (7.6)
Non-White	9.2%	5.4%	7.8%	6.5%	9.9%	4.9%
Smoker	23.1%	29.7%	26.4%	36.2%	21.5%	26.6%
BMI	28.2 (5.6)	28.0 (5.9)	28.2 (4.6)	27.4 (4.9)	28.2 (6.1)	28.3 (6.4)
SBP	132.6 (16.3)	130.1 (16.0)	135.6 (16.1)	130.3 (14.5)	131.2 (16.3)	130.0 (16.6)
Total cholesterol	5.4 (1.1)	5.5 (1.1)	5.3 (1.1)	5.3 (1.1)	5.5 (1.1)	5.5 (1.1)
HDL	1.5 (2.8)	1.5 (0.5)	1.3 (1.0)	1.4 (0.5)	1.6 (3.3)	1.5 (0.4)
Diabetes	12.2%	13.8%	13.0%	15.0%	11.8%	13.2%

ASCVD = Atherosclerotic Cardiovascular Disease score; QRISK3 = QRESEARCH risk estimator version 3 score; SCORE2 = Systematic Coronary Risk Evaluation version 2 score; PwMS = people with Multiple Sclerosis; SBP = systolic blood pressure; HDL = high-density lipoprotein.

### Discrimination performance of the VRCs

**Figure 1.**
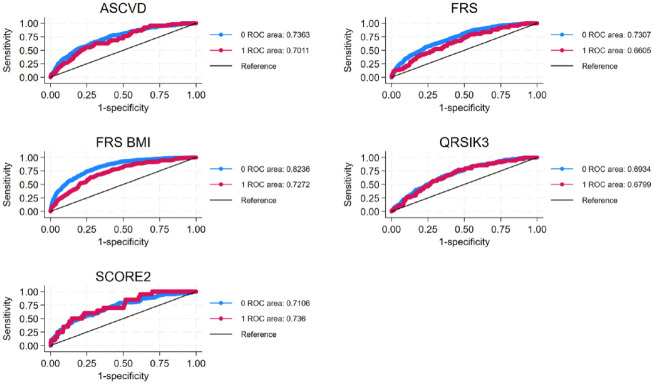
ROC areas for the vascular risk calculators in each derivation sub-cohort. Blue line indicates the ROC areas for the general population, while red line indicates the ROC areas for people with MS. ASCVD = Atherosclerotic Cardiovascular Disease score; FRS = Framingham Risk Score; FRS-BMI = BMI-based Framingham Risk Score; QRISK3 = QRESEARCH risk estimator version 3 score; SCORE2 = Systematic Coronary Risk Evaluation version 2 score; ROC = Receiver Operating Characteristic.

VRCs performed well when considering the continuous score and the area under the ROC curve (AUROC, Figure 1). Overall, several VRCs demonstrated moderate-to-good discrimination in the MS and control groups. For example, the ASCVD, FRS-BMI and SCORE2 achieved AUROC > 0.70 in PwMS and controls. QRISK3 and Framingham (cholesterol-based) models showed more modest discrimination, with an AUROC of 0.57–0.66, with slightly lower performance among PwMS than controls.

However, performance was poor when standard clinical cut-points were applied (Table 2). Sensitivity was poor across all models in PwMS, whereas specificity tended to be higher. For instance, at the ASCVD 7.5% threshold, sensitivity and specificity in PwMS were 58.2% and 70.4%, respectively (AUROC approximation: 0.64). QRISK3 showed a similar pattern, with 60.9% sensitivity and 64.8% specificity (AUROC approximation: 0.63). SCORE2 performed poorly at its recommended threshold, with a sensitivity of 30.0% and specificity of 34.0% in PwMS (AUROC approximation: 0.32).

**Table 2.** Summary statistics for the cut-point for intervention for each vascular risk calculator.

ASCVD																		
	PwMS									Controls								
	Everybody			Males			Females			Everybody			Males			Females		
	Value	95% CI		Value	95% CI		Value	95% CI		Value	95% CI		Value	95% CI		Value	95% CI	
Sensitivity	58.20	45.50	70.20	71.40	51.30	86.80	48.70	32.40	65.20	68.30	62.60	73.60	84.70	77.00	90.70	57.10	49.50	64.60
Specificity	70.40	68.10	72.60	48.10	43.70	52.60	80.60	78.20	82.90	65.40	64.40	66.50	43.00	41.10	45.00	75.70	74.50	76.80
ROC area	0.64	0.58	0.7	0.6	0.51	0.69	0.65	0.57	0.73	0.67	0.64	0.7	0.64	0.61	0.67	0.66	0.63	0.7
PPV	7.50	5.40	10.10	7.00	4.30	10.60	8.10	4.90	12.30	6.60	5.70	7.50	6.40	5.20	7.70	6.80	5.60	8.20
NPV	97.60	96.60	98.40	96.90	93.90	98.60	97.80	96.70	98.70	98.30	97.90	98.60	98.40	97.50	99.10	98.30	97.80	98.60
Framingham																		
	PwMS									Controls								
	Everybody			Males			Females			Everybody			Males			Females		
	Value	95% CI		Value	95% CI		Value	95% CI		Value	95% CI		Value	95% CI		Value	95% CI	
Sensitivity	26.90	16.80	39.10	40.70	22.40	61.20	17.50	7.30	32.80	43.30	37.30	49.40	64.20	54.30	73.20	30.20	23.40	37.70
Specificity	87.20	85.60	88.60	73.00	69.30	76.50	93.60	92.20	94.80	84.60	83.90	85.30	67.80	66.10	69.60	92.30	91.60	92.90
ROC area	0.57	0.52	0.62	0.57	0.47	0.66	0.56	0.5	0.62	0.64	0.61	0.67	0.66	0.61	0.71	0.61	0.58	0.65
PPV	6.60	4.00	10.30	6.20	3.10	10.80	7.40	3.00	14.70	7.80	6.50	9.20	6.80	5.40	8.60	9.40	7.10	12.20
NPV	97.20	96.40	97.90	96.60	94.50	98.00	97.50	96.50	98.30	98.00	97.70	98.30	98.10	97.40	98.60	98.00	97.60	98.40
Framingham BMI																		
	PwMS									Controls								
	Everybody			Males			Females			Everybody			Males			Females		
	Value	95% CI		Value	95% CI		Value	95% CI		Value	95% CI		Value	95% CI		Value	95% CI	
Sensitivity	29.80	23.50	36.90	45.60	33.50	58.10	21.10	14.30	29.40	47.60	43.60	51.60	66.20	59.80	72.20	36.10	31.30	41.10
Specificity	90.10	89.50	90.70	77.70	76.10	79.30	95.30	94.70	95.80	90.80	90.60	91.10	78.90	78.30	79.60	95.90	95.70	96.10
ROC area	0.6	0.57	0.63	0.62	0.56	0.68	0.58	0.55	0.62	0.69	0.67	0.71	0.73	0.7	0.76	0.66	0.64	0.68
PPV	5.80	4.50	7.50	4.80	3.30	6.80	7.80	5.10	11.20	5.30	4.70	5.90	4.20	3.60	4.90	7.70	6.50	9.00
NPV	98.40	98.10	98.70	98.30	97.70	98.80	98.50	98.10	98.80	99.40	99.30	99.40	99.40	99.30	99.5	99.40	99.30	99.40
QRISK3																		
	PwMS									Controls								
	Everybody			Males			Females			Everybody			Males			Females		
	Value	95% CI		Value	95% CI		Value	95% CI		Value	95% CI		Value	95% CI		Value	95% CI	
Sensitivity	60.90	48.40	72.40	44.40	25.50	64.70	71.40	55.40	84.30	67.40	61.80	72.70	53.90	44.40	63.20	76.00	69.10	82.00
Specificity	64.80	62.70	66.90	75.30	71.80	78.60	60.10	57.50	62.60	61.50	60.50	62.50	71.30	69.60	72.90	57.10	55.90	58.30
ROC area	0.63	0.57	0.69	0.6	0.5	0.7	0.66	0.59	0.73	0.64	0.62	0.67	0.63	0.58	0.67	0.67	0.63	0.7
PPV	5.40	3.90	7.20	7.00	3.70	11.90	5.00	3.40	7.00	5.10	4.50	5.90	6.70	5.20	8.50	4.70	3.90	5.50
NPV	98.00	97.20	98.70	97.00	95.10	98.30	98.60	97.60	99.30	98.40	98.00	98.70	97.60	96.80	98.20	98.90	98.50	99.20
SCORE2																		
	PwMS									Controls								
	Everybody			Males			Females			Everybody			Males			Females		
	Value	95% CI		Value	95% CI		Value	95% CI		Value	95% CI		Value	95% CI		Value	95% CI	
Sensitivity	30.00	11.90	54.30	10.00	0.30	44.50	50.00	18.70	81.30	35.00	26.50	44.20	12.50	4.70	25.20	50.00	38.00	62.00
Specificity	34.00	30.60	37.50	58.90	52.30	65.20	22.40	18.80	26.30	37.30	36.00	38.70	64.60	62.30	66.90	24.10	22.70	25.60
ROC area	0.32	0.22	0.42	0.34	0.24	0.45	0.36	0.2	0.53	0.36	0.32	0.4	0.39	0.34	0.43	0.37	0.31	0.43
PPV	1.20	0.40	2.60	1.00	0.00	5.60	1.30	0.40	2.90	1.30	0.90	1.70	1.00	0.40	2.20	1.40	0.90	1.90
NPV	94.70	91.30	97.10	93.90	88.80	97.20	95.80	90.40	98.60	96.10	95.10	96.90	96.30	95.00	97.30	95.90	94.30	97.10

ASCVD = Atherosclerotic Cardiovascular Disease score; QRISK3 = QRESEARCH risk estimator version 3 score; SCORE2 = Systematic Coronary Risk Evaluation version 2 score; PwMS = people with Multiple Sclerosis; ROC = Receiver Operating Characteristic; PPV = positive predictive value; NPV = negative predictive value.

### Recalibration of vascular risk calculators

Of the five VRCs tested, only the FRS-BMI equation retained all statistically significant predictors (*p* < 0.05) in the Cox regression and proceeded to recalibration (see Supplemental Table 2).

For the cross-validated recalibrated FRS-BMI model with the original predictors and MS status as an additional covariate (model A), the Somers’ D coefficient was 0.815 (95% CI = 0.801–0.829) (Table 3). The model (B) that incorporated interaction terms between MS and all predictors performed similarly (Somers’ D coefficient: 0.817 (95% CI = 0.803–0.831)) to model A (*p* = 0.054). When comparing the two newly derived models with the FRS-BMI equation, both models showed significant gain in discriminative performance (Model A: Δ Somers’ D = 0.023, 95% CI = 0.017–0.030; Model B: Δ Somers’ D = 0.026, 95% CI = 0.018–0.033). Calibration, assessed via Hosmer–Lemeshow-type chi-square statistics, showed excellent fit (Model A: χ^2^ = 74,006; Model B: χ^2^ = 77,226). Although Models A and B demonstrated comparable performance regarding discrimination and calibration, we selected Model A for parsimony.

**Table 3.** Cox model for the recalibration of the Framingham Score with BMI.

	Model A				Model B			
	Coeff.	95% CI		*p*	Coeff.	95% CI		*p*
MS	0.5546	0.3912	0.7181	<0.001	5.24	1.93	8.56	0.002
ln (age)	3.58	3.19	3.98	<0.001	3.86	3.4	4.32	<0.001
Male	0.17	0.026	0.31	0.021	0.17	0.03	0.32	0.010
ln (BMI)	0.7	0.26	1.14	0.002	0.67	0.23	1.11	0.003
ln (SBP)	1.47	0.78	2.16	<0.001	1.44	0.75	2.13	<0.001
Antihypertensive treatment	0.73	0.55	0.9	<0.001	0.85	0.66	1.05	<0.001
Smoker	0.7	0.56	0.84	<0.001	0.79	0.63	0.95	<0.001
Diabetes	1.3	1.13	1.46	<0.001	1.29	1.12	1.46	<0.001
MS##ln(age)					−1.1	−1.93	−0.26	0.010
MS##antihypertensive treatment					−0.51	−0.91	−0.1	0.015
MS##smoker					−0.4	−0.73	−0.07	0.019

The model A did not consider interaction terms for Multiple Sclerosis (MS), while the model B considered interaction terms for MS. BMI = body mass index; SBP = systolic blood pressure; ln = natural log.

For the selected model, an optimal cut-point for intervention was 2.0% (sensitivity: 69.7%, specificity: 73.1%, correctly classified: 73.1%). Applying this cut-point to the PwMS in the calibration cohort, 38.8% of this population would be considered high risk.

### Baseline characteristics of the validation cohort

The validation cohort comprised 324,386 individuals, including 45,934 who were PwMS; 69.2% were females (Table 4). Average age at index year was 44 years across all groups, while the proportion of the population that was non-White population ranged from 1% to 2%. Smoking was consistently higher in PwMS (23.9% in females with MS), while BMI was consistently higher in controls as compared with PwMS (controls: 26.1, SD: 4.7; PwMS: 25.8, SD: 4.8). However, diabetes prevalence was consistently higher in PwMS than in controls, although it never exceeded 3% (3% in males with MS).

**Table 4.** Descriptive statistics of the validation cohort for the recalibrated Framingham BMI.

Framingham BMI (validation cohort)						
	Both sexes		Males		Females	
	Matched controls	PwMS	Matched controls	PwMS	Matched controls	PwMS
*N*	278,452	45,934	85,878	14,004	192,574	31,930
Score	6.9 (7.9)	6.9 (7.7)	11.0 (10.2)	11.0 (10.1)	5.1 (5.7)	5.1 (5.5)
Age	44.3 (10.2)	44.2 (10.1)	44.8 (10.2)	44.7 (10.2)	44.1 (10.2)	44.0 (10.2)
Non-White	1.8%	0.9%	1.8%	1.1%	1.8%	0.8%
Smoker	17.0%	21.0%	18.5%	23.9%	16.3%	19.7%
BMI	26.1 (4.7)	25.8 (4.8)	26.5 (4.0)	25.8 (4.0)	25.9 (5.0)	25.8 (5.1)
SBP	127.8 (16.4)	126.5 (15.8)	131.9 (15.0)	129.8 (14.7)	125.9 (16.6)	125.0 (16.0)
Antihypertensive treatment	4.5%	6.4%	4.4%	6.1%	4.6%	6.6%
Diabetes	1.6%	2.4%	2.0%	3.0%	1.4%	2.2%
Index of multiple deprivation						
1, least deprived	19.0%	19.1%	18.5%	18.5%	19.3%	19.4%
2	18.0%	18.1%	17.7%	17.8%	18.2%	18.3%
3	22.5%	22.5%	22.1%	22.2%	22.7%	22.6%
4	21.1%	21.1%	21.5%	21.5%	20.9%	20.8%
5, most deprived	19.4%	19.4%	20.3%	20.3%	19.0%	18.8%

PwMS = people with Multiple Sclerosis.

### External validation of the recalibrated FRS-BMI risk equation

In the external validation cohort, the recalibrated FRS-BMI score showed a good discriminating ability (Somers’ D = 0.716, 95% CI = 0.710–0.716). When comparing the newly derived score with the original FRS-BMI equation, the score showed a marginal gain in discriminative performance in the validation data set as well (Δ Somers’ D = 0.003, 95% CI = 0.002–0.003; Supplemental Figure 2).

## Discussion

We comprehensively evaluated and recalibrated widely used VRCs for PwMS, using a large, population-based data set. While several VRCs (including ASCVD, SCORE2 and FRS-BMI) demonstrated moderate-to-good discrimination when used as continuous scores, their performance was substantially reduced at clinically relevant decision thresholds. Importantly, only the FRS-BMI score retained statistical validity across all terms when applied to the MS population and was recalibrated.

After model extension with the addition of MS status, the revised FRS-BMI model demonstrated improved discrimination (Δ Somers’ D = 0.023–0.026) and retained good calibration. The principle of parsimony supported the selection of this model (without interaction terms between MS and other terms), enhancing interpretability and potential for clinical implementation without compromising predictive accuracy. However, external validation revealed only a modest performance gain over the original model (Δ Somers’ D = 0.003), indicating that the clinical utility of the recalibrated model remains limited. These findings show that the risk factors included in existing models are insufficient, and *de novo* derivation of an MS-specific vascular risk algorithm is needed to achieve clinically meaningful advances in vascular risk stratification. Future studies should evaluate whether the recalibrated model improves treatment decisions in practice, for example, using decision curve analysis across clinically relevant thresholds and reclassification measures centred on guideline-based thresholds such as those for statin therapy.^32^ We note that recent studies suggest that biological age and frailty may be important indices in predicting cardiovascular events, both observations that may be relevant to the MS population.^33^

A key strength of our study lies in the large, population-based nature of both calibration and validation cohorts, encompassing more than 300,000 individuals, with consistent MS case definitions and follow-up through linked electronic health record data. The methodological rigour applied in calibration enhances internal validity and reproducibility. Unlike previous studies of model recalibration (e.g. in osteoporosis), our approach explicitly considered model extension, which has been recognized as a best practice when significant shifts in underlying risk are expected.^29,30^ The long study window spanned substantial evolution in MS diagnosis and management, and in cardiovascular prevention;^34,35^ we did not model these temporal changes explicitly as this was outside our scope, although prior population-based work showed consistent excess macrovascular risk after adjustment across eras.^1^

Nevertheless, several limitations warrant discussion. First, although our recalibrated model showed statistical improvement, the incremental gain in clinical discrimination was small. This may reflect the mismatch between the assumptions embedded in general-population models and the pathophysiological profile of PwMS, including systemic inflammation, neurovascular interactions and differential medication use, which likely influences vascular risk in ways not captured by traditional predictors. Second, high and selective missingness of lipid measurements precluded reliable imputation, likely penalizing cholesterol-based equations over BMI-based ones; this limitation may have contributed to the apparent superiority of FRS-BMI, partly reflecting data availability rather than true model fitness. Also, some DMTs used in MS can affect lipid profiles,^36^ highlighting the importance of lipid capture in future data sets. Third, our approach did not explore non-linear interactions. Fourth, including a binary variable for the MS status does not capture relevant MS-specific characteristics such as disease duration, phenotype or treatment exposure, but reflects the intended use in primary care settings as well, where such information is often unavailable. Fifth, by design, we constrained predictors to those in the original tools plus MS status. Therefore, other variables, including alcohol consumption, that can relate to white matter changes and cerebrovascular risk,^37^ were not included. Similarly, there may be some residual confounding from variables such as physical activity. These may be considered in a future MS-specific calculator. Furthermore, neuroimaging data were not available, and acute demyelinating lesions in MS can rarely mimic ischemic stroke on diffusion-weighted imaging.^38,39^ However, in large data sets using linked hospital and mortality data, any resulting misclassification is likely limited and may be bidirectional, tending to bias estimates towards the null.^21,40^ Finally, although the inclusion of MS status and its interactions improves statistical fit, it does not address potential mediators (e.g. corticosteroid use and frailty status), which may drive excess vascular risk. Future studies should aim to incorporate such MS-specific factors directly into prediction algorithms.

These findings have tangible implications for vascular risk management in MS. First, even a modestly improved score, such as the recalibrated FRS-BMI, could serve as an interim tool for better identifying at-risk individuals pending the development of new calculators. Importantly, current international guidelines for cardiovascular prevention do not include MS as a high-risk condition.^11^ Given the consistent evidence of increased vascular risk in PwMS, especially at younger ages and in women, our results support reclassification of MS as a vascular risk-enhancing condition, analogous to rheumatoid arthritis. Second, disease-specific guidelines should consider incorporating vascular risk scoring tools adapted for PwMS, particularly at MS diagnosis. Risk scores could guide targeted prevention, including lipid-lowering therapy, blood pressure control and smoking cessation – interventions which are underutilized in this population despite their proven benefit.^6^

Last, researchers and policymakers should prioritize prospective derivation of MS-specific vascular risk models using multi-country electronic health records or registry data sets, potentially incorporating widely available biomarkers or neuroimaging metrics to capture vascular contributions unique to MS pathogenesis.

## Conclusion

In summary, we evaluated five established VRCs in a large MS cohort, finding modest clinical improvement after statistical recalibration in the FRS-BMI. The modest gain in predictive performance following model extension suggests that recalibrating general-population models is insufficient for addressing the vascular risk burden in PwMS. Development of new, MS-specific VRCs appears necessary to enable personalized, preventive vascular care in this population.

## Supplemental Material

sj-docx-1-msj-10.1177_13524585261444243 – Supplemental material for Recalibration and external validation of vascular risk calculators for multiple sclerosis: A population-based study using CPRD data in England, 1987–2023 Supplemental material, sj-docx-1-msj-10.1177_13524585261444243 for Recalibration and external validation of vascular risk calculators for multiple sclerosis: A population-based study using CPRD data in England, 1987–2023 by Raffaele Palladino, Ruth Ann Marrie, Giuseppina Affinito, Azeem Majeed, Stephen Allan Schaffer and Jeremy Chataway in Multiple Sclerosis Journal
