# Supplementary material for: Recalibration and external validation of vascular risk calculators for multiple sclerosis: A population-based study using CPRD data in England, 1987–2023
Source: Mult Scler. 2026 May 12;32(8):806–17. doi: 10.1177/13524585261444243 (PMC13333034; doi:10.1177/13524585261444243)

**Supplemental Material**

**Recalibration and external validation of Vascular Risk Calculators for Multiple Sclerosis: a Population-based study using CPRD data in England, 1987-2023.**

Raffaele Palladino^1,2^, Ruth Ann Marrie^3,4^, Giuseppina Affinito^2^, Azeem Majeed^1^, Stephen Allan Schaffer^3^, Jeremy Chataway^6,7^

1. Department of Primary Care and Public Health, School of Public Health, Imperial College of London, London, United Kingdom.

2. Department of Public Health, Federico II University, Naples, Italy

3. Department of Internal Medicine, Max Rady College of Medicine, Rady Faculty of Health Sciences, University of Manitoba, Winnipeg, Can.

4. Departments of Medicine and Community Health and Epidemiology, Faculty of Medicine, Dalhousie University, Halifax, Canada. Nova Scotia Health, Halifax, Canada

5.Queen Square Multiple Sclerosis Centre, Department of Neuroinflammation, UCL Queen Square Institute of Neurology, Faculty of Brain Sciences, University College London, London, United Kingdom WC1N 3BG

6. National Institute for Health and Care Research, University College London Hospitals Biomedical Research Centre, London, United Kingdom

WC1B 5EH

**EXTENDED METHODS**

We conducted a population-based study of PwMS and matched controls registered with general practices in England, from 1987 to 2023. The study used data from routinely collected electronic health records from two Clinical Practice and Research Datalink (CPRD) databases. Ethics approval was granted by the Independent Scientific Advisory Committee of the CPRD (protocol number: 22_002520 and 18_279R).

**Data source**

We used two of the largest UK electronic health record databases: CPRD GOLD (1986-2018) and CPRD Aurum (1990-2023), which hold anonymised, routinely collected longitudinal primary care records ^1, 2^. They are representative in terms of socio-demographic characteristics ^1-4^. The *calibration* cohort was drawn from CPRD GOLD, which includes data from general practices using the Vision® software system. The database covered approximately 7% of the UK population in 2018 ^1, 3^. The *validation* cohort was drawn from CPRD Aurum, which includes data from practices using EMIS® software and covered approximately 24% of the English population as of December 2023. For both data sources we limited the analysis to English practices^1^ as primary care data are linked to multiple sources, including Hospital Episode Statistics (HES), Office for National Statistics (ONS) mortality, and deprivation data, using NHS numbers.

**Study population**

MS cases were identified using a previously described algorithm.^5, 6^ Briefly, possible MS cases were identified based on ICD-X codes, diagnostic and management primary care codes (Read codes for CPRD Gold and SNOMED-CT and Read codes for CPRD Aurum), and on prescription of disease-modifying therapies specific to MS.^7^ In line with previous work ^8^, PwMS were identified as those with ≥3 MS events recorded in their available clinical history, to reduce the risk of misclassification. The index date was considered as the date of the first MS diagnosis ^6^.

In line with previously published work, additional inclusion criteria for MS cases were: (i) diagnosis after 1-Jan 1987 for CPRD Gold and 1-Jan 1990 for CPRD Aurum, (following the introduction of MRI to support the diagnosis); (ii) individuals were required to have uninterrupted CPRD practice registration for ≥1 year before the first MS event to ensure that key covariate information was available at the index date; (iii) available information on sex (male/female); (iv) presence of a valid date of birth; (v) age ≥18 years at index date; (vi) MS events recorded prior the date of death; and (vii) validity of patients’ clinical records with respect to continuous follow-up and data entry, as defined by the CPRD’s up-to-standard (UTS) criteria (only available for CPRD Gold) ^6^.

To enhance sample size, we included up to two time points per participant: data from the index year and, when available, data collected at age 50 (±5 years). This age range was selected given that vascular risk assessments are commonly performed around age 50 years in England ^9^.

To assess the discriminative performance of the VRCs, PwMS were randomly matched to as many as six individuals without MS based on general practice, sex, and age. Controls were required to have UTS clinical data available during the study period (CPRD Gold only) and to have no recorded diagnosis of MS or any other demyelinating condition (e.g., optic neuritis, transverse myelitis, acute disseminated encephalomyelitis, or central nervous system demyelination not otherwise classified); this reduced the likelihood of including individuals who might later develop MS. Multiple controls were matched to each PwMS to decrease variance.^10^ We assigned the controls the index date of their matched MS case.

As VRCs estimate the 10-year risk of cardiovascular disease (CVD) or CVD-related mortality, we excluded individuals with a history of these outcomes before the index date, tailoring the inclusion criteria to match the specific definitions of CVD and applicable age ranges used by each calculator (see Supplemental Table 1).

**Study variables**

All variables considered for the present study included: age; sex; ethnicity; index of multiple deprivation;^11^ BMI; smoking status; systolic blood pressure and a measure of systolic blood pressure variability; total cholesterol and total cholesterol/HDL ratio; diagnosis of the following chronic diseases: diabetes, chronic renal disease, migraine, lupus, and severe mental illness; treatment with antihypertensive medication, corticosteroid, and atypical antipsychotics; and family history of macrovascular disease. Outcomes included 10-year risk of CVD and CVD-mortality. As part of the CVD definition (see Supplementary Table 1 for details) cerebrovascular disease was identified from CPRD, HES, and ONS records using validated, primary care records, stroke‑specific ICD‑10 codes in hospital data and cause‑of‑death codes where applicable, an approach that improves completeness and accuracy of stroke capture in English routine data ^12-14^. Supplemental Table 1 summarises the variables included in each VRC.

**Statistical analysis**

PwMS with missing data on smoking at index were classified as non-smokers, if there was no prior indication of smoking history ^15, 16^. Those missing data on ethnicity were classified as White ^17^. For BMI, systolic blood pressure, total cholesterol, and HDL, if data were missing at index year, we considered records in the following and previous five years ^6, 15^. Consistent with previous studies using CPRD data in MS research ^5, 6, 18, 19^ we employed the multiple imputation method to handle missing data for time-varying variables including BMI, systolic blood pressure, total cholesterol, and HDL. After checking the missing data percentage for these variables and multiple imputation assumptions, we employed multiple imputation by chained equation (10 copies) and combined results using Rubin’s rules for BMI and systolic blood pressure (calibration cohort: 49.9% for blood pressure and 50% for BMI; validation cohort: 24.9% for blood pressure and 25.7% for BMI)^20^. Given the high percentage of missing data, multiple imputation was not possible for cholesterol data. The regression model for the multiple imputation included these independent variables: sex, age, ethnicity, smoking status in the index year, number of comorbidities (considering those included in FRS, FRS-BMI, SCORE2, ASCVD, and QRISK3)^21-23^ in the index year, treatment with lipid-lowering, oral anti-diabetic, anti-platelet, anti-coagulant, and anti-hypertensive therapies in the index year, number of visits in the previous year, region, and index of multiple deprivation (quintiles) ^11^.

The recalibration and validation process comprised several steps. First, we assessed the performance of the original risk equation employed for the VRCs in the calibration cohort by calculating the receiver operating characteristic (ROC) curve area using the Youden index approximation, considering the continuous score and the binary variable defined by the published cut-point for intervention (binary classifier) for each VRC. Second, for ROC <0.70 for PwMS, we recalibrated each VRC, which involves adjusting baseline hazard functions and population-specific means to better reflect the target group, an approach applied elsewhere ^24^. Specifically, we employed Cox regression models including the terms of the original equations as independent variables and added MS status (binary variable) as an additional term ^25^. We limited model extension to the inclusion of an MS indicator to preserve real-world applicability since our recalibration was designed for implementation using routinely collected data also in primary care settings where MS-specific measures such as phenotype, disability status, inflammatory activity, and detailed DMT exposures are not consistently available. Transformations applied to continuous variables in the original VRCs were preserved during the recalibration and incorporated into the Cox model accordingly. Although we refer to this process as "recalibration," it more accurately reflects a model revision approach. Specifically, we extended the original VRC by including MS status as an additional predictor; this constitutes a hybrid between recalibration and derivation ^26, 27^.

Third, the recalibration process was conducted only for risk equations in which all terms remained statistically significant (p < 0.05) in the Cox model to ensure the reliability and interpretability of the recalibrated coefficients. Including non-significant predictors would have resulted in unstable or non-reproducible point estimates, potentially introducing bias or reducing generalizability ^26, 27^. As previously suggested, we evaluated the calibration of the derived risk algorithm in our internal dataset by stratifying the cohort by deciles of risk score and employing a Hosmer-Lemeshow test.^21^ Finally, k-fold (10 folds) cross-validation was employed to calculate the Somers’ d statistics.^28^ For VRCs that were successfully recalibrated, an interaction term between MS and other terms was also tested. The recalibrated VRCs were tested externally using the validation and calculating the Somers’ d statistics ^28^; for completeness, the AUROC and ROC curve for the externally validated score were additionally reported in the Supplementary Material.

**REFERENCES**

1. CPRD. Clinical Practice Research Datalink.

2. CPRD. CPRD Aurum December 2023 dataset. 2023.

3. Mathur R, Bhaskaran K, Chaturvedi N, et al. Completeness and usability of ethnicity data in UK-based primary care and hospital databases. *J Public Health (Oxf)*. 2014; 36: 684-92.

4. Wolf A, Dedman D, Campbell J, et al. Data resource profile: Clinical Practice Research Datalink (CPRD) Aurum. *Int J Epidemiol*. 2019.

5. Palladino R, Chataway J, Majeed A and Marrie RA. Interface of Multiple Sclerosis, Depression, Vascular Disease, and Mortality: A Population-Based Matched Cohort Study. *Neurology*. 2021; 97: e1322-e33.

6. Palladino R, Marrie RA, Majeed A and Chataway J. Evaluating the risk of macrovascular events and mortality among people with multiple sclerosis in England. *Jama Neurology*. 2020; 77: 820-8.

7. CPRD. Primary care data for public health research.

8. Culpepper WJ, Marrie RA, Langer-Gould A, et al. Validation of an algorithm for identifying MS cases in administrative health claims datasets. *Neurology*. 2019; 92: e1016-e28.

9. Chang KC, Vamos EP, Palladino R, Majeed A, Lee JT and Millett C. Impact of the NHS Health Check on inequalities in cardiovascular disease risk: a difference-in-differences matching analysis. *J Epidemiol Community Health*. 2019; 73: 11-8.

10. Stuart EA. Matching methods for causal inference: A review and a look forward. *Stat Sci*. 2010; 25: 1-21.

11. GOV.UK. English indices of deprivation 2015.

12. Morgan A, Sinnott SJ, Smeeth L, Minassian C and Quint J. Concordance in the recording of stroke across UK primary and secondary care datasets: a population-based cohort study. *BJGP Open*. 2021; 5: BJGPO.2020.0117.

13. McCormick N, Bhole V, Lacaille D and Avina Zubieta JA. Validity of diagnostic codes for acute stroke in administrative databases: a systematic review. *PLoS One*. 2015; 10: e0135834.

14. Li L, Binney LE, Luengo Fernandez R, Silver LE and Rothwell PM. Temporal trends in the accuracy of hospital diagnostic coding for identifying acute stroke: a population-based study. *European Stroke Journal*. 2020; 5: 26-35.

15. Palladino R, Tabak AG, Khunti K, et al. Association between pre-diabetes and microvascular and macrovascular disease in newly diagnosed type 2 diabetes. *BMJ Open Diabetes Res Care*. 2020; 8.

16. Palladino R, Vamos EP, Chang KC, Khunti K, Majeed A and Millett C. Evaluation of the Diabetes Screening Component of a National Cardiovascular Risk Assessment Programme in England: a Retrospective Cohort Study. *Sci Rep*. 2020; 10: 1231.

17. Mathur R, Bhaskaran K, Chaturvedi N, et al. Completeness and usability of ethnicity data in UK-based primary care and hospital databases. *Journal of Public Health (United Kingdom)*. 2014; 36: 684-92.

18. Palladino R, Marrie RA, Majeed A and Chataway J. Management of vascular risk in people with multiple sclerosis at the time of diagnosis in England: A population-based study. *Mult Scler*. 2023; 29: 671-9.

19. Palladino R, Chataway J, Mathew M, Majeed A and Marrie RA. Trajectories and management of vascular risk following the diagnosis of multiple sclerosis: A population-based matched cohort study between 1987 and 2018 in England. *Mult Scler*. 2024; 30: 1653-63.

20. Rubin DB. *Multiple imputation for nonresponse in surveys*. New York ;: Wiley, 1987.

21. D'Agostino RB, Vasan RS, Pencina MJ, et al. General cardiovascular risk profile for use in primary care: The Framingham heart study. *Circulation*. 2008; 117: 743-53.

22. Europe CfPGtitqocpapci. CVD Prevention - Joint European Guidelines on Cardiovascular Disease Prevention in Clinical Practice. European Society of Cardiology, 2016.

23. National Institute for Health and Care Excellence. Cardiovascular disease: risk assessment and reduction, including lipid modification. London, the UK: National Institute for Health and Care Excellence, 2014.

24. Bisson EJ, Finlayson ML, Ekuma O, Marrie RA and Leslie WD. Accuracy of FRAX ® in People With Multiple Sclerosis. *Journal of Bone and Mineral Research*. 2019; 34: 1095-100.

25. DeFilippis AP, Young R, Carrubba CJ, et al. An analysis of calibration and discrimination among multiple cardiovascular risk scores in a modern multiethnic cohort. *Ann Intern Med*. 2015; 162: 266-75.

26. Steyerberg EW, Vickers AJ, Cook NR, et al. Assessing the Performance of Prediction Models. *Epidemiology*. 2010; 21: 128-38.

27. Moons KG, Kengne AP, Woodward M, et al. Risk prediction models: I. Development, internal validation, and assessing the incremental value of a new (bio)marker. *Heart*. 2012; 98: 683-90.

28. Newson RB. Comparing the Predictive Powers of Survival Models Using Harrell's C or Somers’ D. *The Stata Journal*. 2010; 10: 339-58.

29. American Diabetes A. 10. Cardiovascular Disease and Risk Management: Standards of Medical Care in Diabetes-2020. *Diabetes Care*. 2020; 43: S111-S34.

30. Arnett DK, Blumenthal RS, Albert MA, et al. 2019 ACC/AHA Guideline on the Primary Prevention of Cardiovascular Disease: Executive Summary: A Report of the American College of Cardiology/American Heart Association Task Force on Clinical Practice Guidelines. *Circulation*. 2019; 140: e563-e95.

**Supplemental Figure 1.** Selection criteria and sample sizes of the study sub-cohorts.

**Notes**: ASCVD = Atherosclerotic Cardiovascular Disease score, QRISK3 = QRESEARCH risk estimator version 3 score, SCORE2 = Systematic Coronary Risk Evaluation version 2 score.


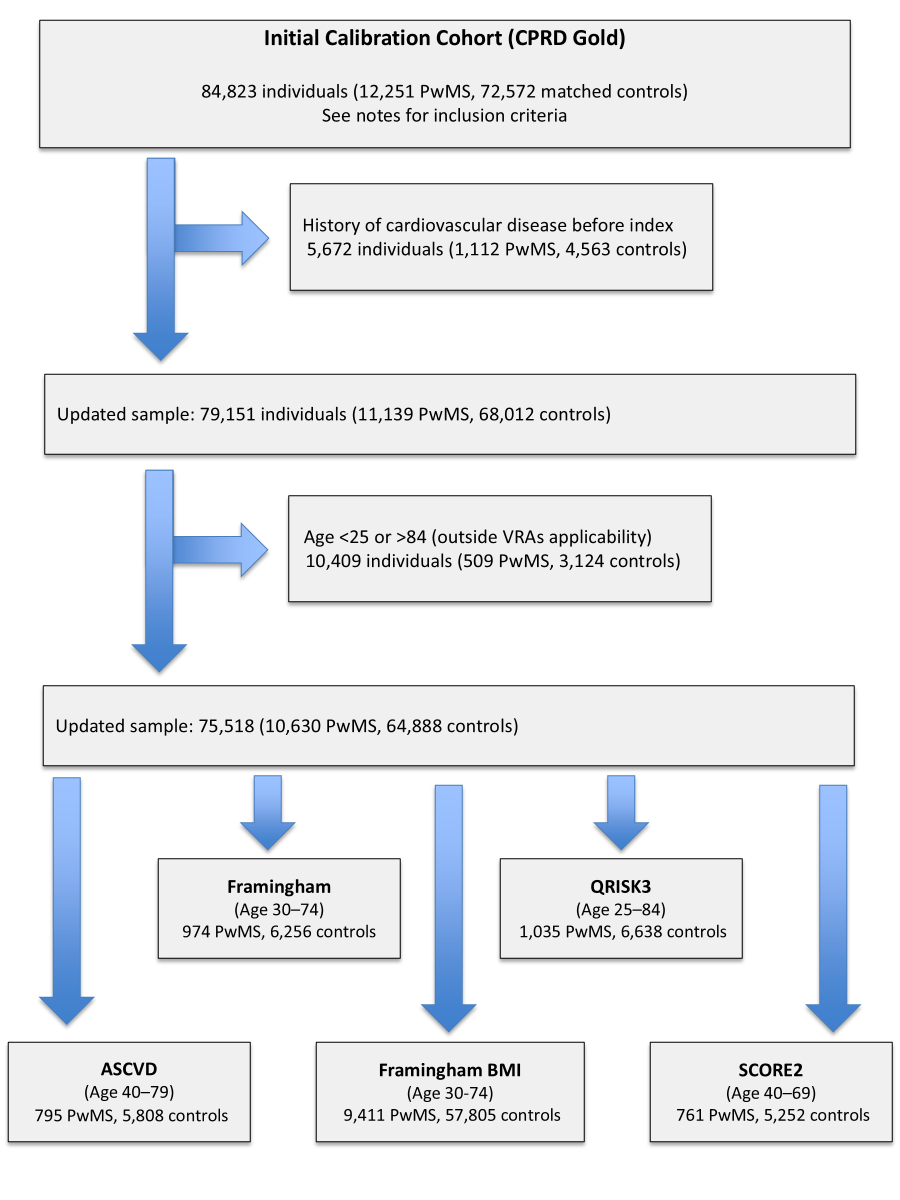


**Supplemental Table 1.** Characteristics of commonly used vascular risk calculators

**Notes**: ASCVD = Atherosclerotic Cardiovascular Disease score, QRISK3 = QRESEARCH risk estimator version 3 score, SCORE2 = Systematic Coronary Risk Evaluation version 2 score.

| **Tool** | **Framingham** | **SCORE** | **QRISK 1-3** | **ASCVD** |
| --- | --- | --- | --- | --- |
| **Country of origin** | United States (Framingham Mass) | 11 European countries | United Kingdom | United States |
| **Calculates** | 10-year risk of cardiovascular disease (CVD) events (original versions included coronary heart disease; 2008 version added  cerebrovascular events, peripheral artery disease, heart failure) | 10-year risk of CVD mortality (death due to cardiovascular disease) | 10-year risk of CVD events (heart attack or stroke) | 10-year risk of atherosclerotic cardiovascular disease |
| **Age range** | 30-75 | 40-65 | 25-84 | 40-79 |
| **Variables considered** | Gender, age, total cholesterol, HDL cholesterol, systolic blood pressure (SBP), smoking status, diabetes, hypertensive treatment | Gender, age, total cholesterol or total cholesterol/HDL ratio, SBP, smoking status | QRISK3: Gender, age, total cholesterol to HDL cholesterol ratio, SBP, measure of SBP variability, smoking status, diabetes, area- based index of deprivation, family history, BMI, antihypertensive treatment, ethnicity, chronic diseases (diabetes: chronic renal disease, migraine, lupus, severe mental illness, erectile dysfunction), and treatments (corticosteroid use, atypical antipsychotics) | Gender, age, race/ethnicity, total and HDL-cholesterol levels, SBP, treatment for hypertension, history of diabetes mellitus (ever) and current smoking status (within the past year). |
| **Notes** | Includes version which does not require laboratory values, substituting BMI for lipid measurements |  |  |  |
| **External validation** | Yes, in UK, Frances, China | Norway, Austria, Iceland, Netherlands |  | Yes |
| **Risk cut-off for intervention** | 20%^21^ | 10% (very high risk)^22^ | 10%^23^ | 15%^29, 30^ |

**Supplemental Table 2.** Cox model for the recalibration of the ASCVD, FRS, QRISK3, and SCORE3 vascular risk calculators.

**Notes:** some of the equations terms were dropped in the statistical model due to collinearity. BMI = body mass index, ln = natural log, HDL = high-density lipoproteins.

| **ASCVD** | **coeff.** | **p-value** | **95%CI** | |
| --- | --- | --- | --- | --- |
| MS | 0.14 | 0.296 | -0.21 | 0.40 |
| age | 0.05 | <0.001 | 0.04 | 0.06 |
| female | 0.00 | 0.066 | -0.14 | 0.01 |
| race | -0.84 | 0.106 | -0.48 | 0.08 |
| ln (total cholesterol) | -0.11 | 0.650 | -0.92 | 0.37 |
| ln (HDL) | -0.88 | 0.001 | -0.30 | -0.46 |
| ln (sbp) | 0.93 | 0.042 | 0.03 | 1.83 |
| smoker | 0.69 | <0.001 | 0.48 | 0.89 |
| Hypertension treatment | 0.78 | <0.001 | 0.57 | 1.00 |
| Diabetes | 0.75 | <0.001 | 0.51 | 0.98 |
| **FRS** | **coeff.** | **p-value** | **95%CI** | |
| MS | 0.30 | 0.014 | 0.06 | 0.55 |
| age | 0.05 | <0.001 | 0.04 | 0.06 |
| male | 0.22 | 0.039 | 0.01 | 0.42 |
| ln (total cholsterol) | -0.15 | 0.529 | -0.61 | 0.31 |
| ln (HDL) | -0.65 | <0.001 | -0.97 | -0.32 |
| ln (sbp) | 0.61 | 0.168 | -0.26 | 1.48 |
| Hypertension treatment | 0.71 | <0.001 | 0.49 | 0.92 |
| smoker | 0.56 | <0.001 | 0.36 | 0.76 |
| Diabetes | 0.82 | <0.001 | 0.59 | 1.04 |
| **QRSIK3** | **coeff.** | **p-value** | **95%CI** | |
| age | 0.05 | <0.001 | 0.03 | 0.06 |
| male | 0.54 | <0.001 | 0.29 | 0.79 |
| Atrial Fibrillation | 1.74 | <0.001 | 1.18 | 2.30 |
| Rheumatoid arthritis | 1.02 | 0.026 | 0.12 | 1.91 |
| Chronic kidney disease | 0.84 | 0.003 | 0.28 | 1.40 |
| Hypertension treatment | 0.59 | <0.001 | 0.34 | 0.83 |
| Diabetes | 1.13 | <0.001 | 0.84 | 1.42 |
| BMI | 0.02 | 0.048 | 0.00 | 0.04 |
| ethnicity |  |  |  |  |
| White | -41.76 | <0.001 |  |  |
| Indian | -44.26 | 1.000 |  |  |
| Pakistani | -0.13 | 0.856 | -1.52 | 1.26 |
| Bangladeshi | -43.08 | <0.001 |  |  |
| Other Asian | -0.06 | 0.913 | -1.20 | 1.08 |
| Black Carribean | 0.52 | 0.250 | -0.37 | 1.41 |
| Black African | 0.39 | 0.507 | -0.76 | 1.53 |
| Chinese | -43.88 | <0.001 |  |  |
| Family history of angina/heart attack | 0.21 | 0.155 | -0.08 | 0.51 |
| total cholesterol/HDL | 0.02 | 0.029 | 0.00 | 0.04 |
| sbp | 0.01 | 0.049 | 0.00 | 0.01 |
| sbp SD | 0.01 | 0.571 | -0.02 | 0.04 |
| smoking (no smoker as ref) | |  |  |  |
| light smoker | -0.50 | 0.620 | -2.46 | 1.47 |
| moderate smoker | 0.52 | <0.001 | 0.27 | 0.76 |
| heavy smoker | 0.83 | 0.103 | -0.17 | 1.83 |
| atypical antipsychotic treatment | 0.42 | 0.452 | -0.67 | 1.50 |
| Severe mental illness | 0.51 | 0.358 | -0.58 | 1.60 |
| Erectile function | 0.07 | 0.788 | -0.46 | 0.60 |
| Systemic lupus erythematosus | -42.02 | <0.001 |  |  |
| Regular steroid tablets | 0.29 | 0.130 | -0.09 | 0.67 |
| **SCORE2** | **coeff.** | **p-value** | **95%CI** | |
| MS | 0.29 | 0.180 | -0.14 | 0.72 |
| age | 0.04 | <0.001 | 0.02 | 0.06 |
| male | 0.26 | 0.107 | -0.06 | 0.58 |
| current smoker | 0.54 | 0.001 | 0.22 | 0.86 |
| sbp | 0.01 | 0.022 | 0.00 | 0.02 |
| total cholesterol | 0.04 | 0.532 | -0.09 | 0.17 |
| HDL | -0.26 | 0.150 | -0.62 | 0.09 |
| Diabetes | 1.45 | <0.001 | 1.13 | 1.77 |

**Supplementary Figure 2:** ROC areas for the recalibrated FRS-BMI risk equation in the external validation cohort

**Notes**: Blue line indicates the ROC areas for original FRS-BMI risk equation, whilst red for recalibrated FRS-BMI risk equation. FRS-BMI = BMI-based Framingham Risk Score.


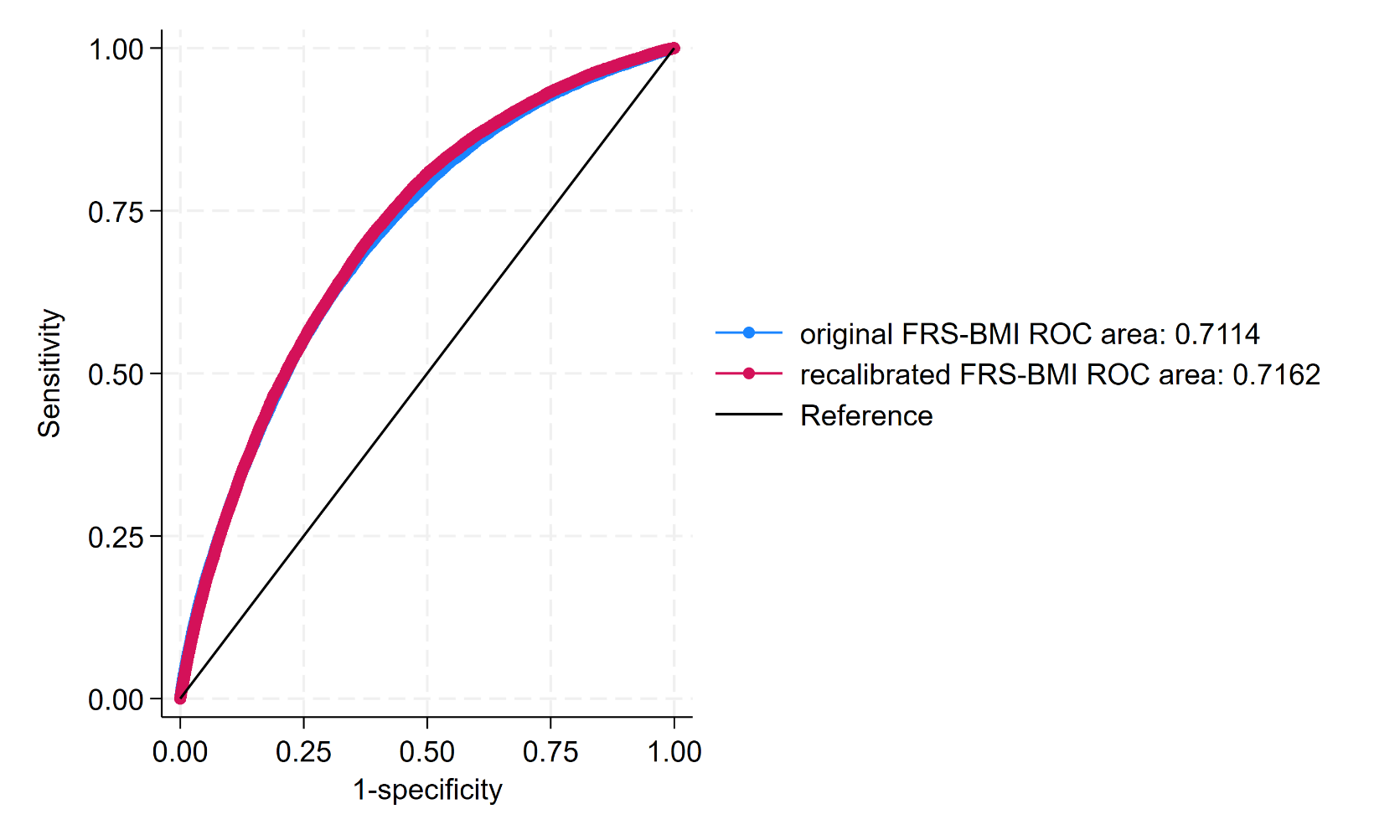

Supplement: sj-docx-1-msj-10.1177_13524585261444243 – Supplemental material for Recalibration and external validation of vascular risk calculators for multiple sclerosis: A population-based study using CPRD data in England, 1987–2023 [file sj-docx-1-msj-10.1177_13524585261444243.docx]
